# Müllerian Cysts of the Posterior Mediastinum: A Case Report and Review of the Literature

**DOI:** 10.7759/cureus.31298

**Published:** 2022-11-09

**Authors:** Christopher Sullivan, Mohammed Amer Swid, James W Klena, Syed A. J. Kazmi, Renee Frank

**Affiliations:** 1 Pathology and Laboratory Medicine, Medical University of South Carolina, Charleston, USA; 2 Pathology and Laboratory Medicine, Geisinger Medical Center, Danville, USA; 3 Surgery, Sentara Mid-Atlantic Cardio-thoracic Surgeons, Norfolk, USA; 4 Anatomic and Clinical Pathology, Geisinger Community Medical Center, Scranton, USA

**Keywords:** cysts, estrogen receptor, mullerian cyst, hattori, mediastinal

## Abstract

Cysts can be segregated according to their embryonic backgrounds. The cysts that were found in the mediastinum are usually divided into bronchogenic cysts, enteric cysts, esophageal cysts, and nonspecific cysts. We add to the relatively small body of literature that exists on this topic by reporting a case of a Müllerian cyst occurring in the posterior mediastinum of a 60-year-old female, showing diffuse nuclear positivity for estrogen receptor (ER) and PAX-8. We examined and summarized the findings of the unique reported cases in the literature. Lastly, an institutional retrospective review of all posterior mediastinal lesions in the last 38.5 years was performed. This revealed that out of 135 candidates within our own healthcare system, the only case consistent with the diagnosis of a mediastinal Müllerian cyst is the report included herein.

## Introduction

Mediastinal cysts are a relatively rare finding and can have a varied and diverse presentation, representing 12%-30% of all mediastinal masses [1]. The most common mediastinal cysts are congenital foregut cysts, which account for roughly 20% of mediastinal masses [2-4]. Meanwhile, neurogenic tumors are the most common cause of a posterior mediastinal mass [4], with about 90% occurring in the posterior mediastinum [5], and comprising 75% of primary posterior mediastinal neoplasms [4, 6]. Of these neurogenic tumors, schwannomas are the most common, frequently presenting as a benign, slow-growing neoplasm that can arise from a spinal or thoracic nerve root [4]. Müllerian cysts, also referred to as Hattori cysts, are a rare pathologic finding typically located in the posterior mediastinum [7-8]. After confirming this cyst to be lined with ciliated non-stratified cuboidal to columnar epithelium, immunohistochemical stains showed positivity for estrogen and progesterone receptors [7-8]. 

With so few reported instances in the literature, Müllerian cysts of the mediastinum are quite rare. However, they can be responsible for common symptoms ranging from discomfort to frank chest pain and shortness of breath with many found incidentally on imaging [9]. Mediastinal Müllerian cysts are considered a benign finding and have no reported recurrence in the literature making surgical excision the treatment of choice [1]. All reported cases occurred in women, the majority of whom were in their perimenopausal period. Associated risk factors such as obesity and/or gynecologic histories such as hormone replacement therapy, hysterectomy, artificial abortion, and oophorectomy were also reported [10]. Herein, we examine the case of a recently diagnosed Müllerian cyst. In order to assess the frequency of these findings, we performed an institutional retrospective review of posterior mediastinal cysts and examined the literature for all instances of cysts with Müllerian differentiation to develop a better understanding of the presentation and epidemiology associated with this finding. 

## Case presentation

**Figure 1:**
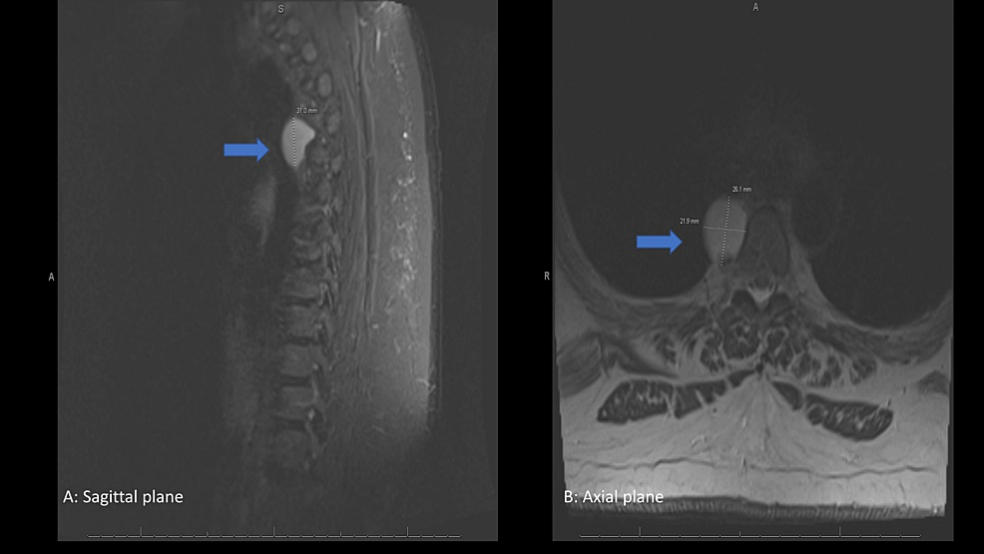
MRI of the thoracic spine. MRI of the thoracic spine showing the cyst (blue arrow) in (A) sagittal and (B) axial plane.

A 60-year-old woman presented with nonspecific complaints of back pain. On CT, a right posterior mediastinal mass was identified. An MRI confirmed an unenhancing right-sided mass, 2.6 cm x 2.2 cm x 3.1 cm, adjacent to the T4 vertebral body (Figure 1). Given the lack of both enhancement and involvement within the neural foramen nerve sheath, a tumor of neural sheath differentiation was unlikely. Findings were suggestive of a neurenteric cyst, duplication cyst, or other benign cystic lesions. Deemed too unstable to undergo surgical excision due to an unrelated medical issue, a six-month follow-up was recommended after which the patient was scheduled for surgical excision. CT performed immediately prior to surgery revealed no interval change in the size or composition of the mass. 

The patient underwent robotic resection of the lesion to rule out malignancy and prevent local symptoms as well as potential malignant degeneration; the whole procedure lasted for almost 2 h. After retraction of the right lung anteriorly, the cyst was easily visualized. It was noted to be in the fourth interspace adjacent to the spine, and just anterior to the sympathetic chain. It was further noted to be adjacent to the fourth intercostal artery and vein. Several clips were utilized for the mobilization of the cyst. Using the Da Vinci robot the posterior mediastinal mass was excised without complication. The estimated blood loss was less than 50 mL. The patient left the operating room with a 20-French right chest tube. The patient received 375 mL of IV crystalloid intraoperatively, and the urine output was 275 mL. The cyst was then completely enucleated, removed, and sent for permanent section. Hemostasis was gained with the use of the bipolar, as well as Surgicel. The patient's right pleural space was irrigated and suctioned dry with a suction irrigator, placed through a 5 mm accessory port. Following completion of this, the patient underwent a T2-T10 thoracic nerve block along each intercostal nerve under direct visualization with the thoracoscope using 2 mL of Exparel at each interspace. The lung was then inflated under direct visualization. The instruments and trocars were removed, and the trocar sites were closed with a layered Vicryl closure with Monocryl for the skin. The chest tube was secured to the skin using a silk suture. The patient tolerated the procedure well and was taken to the post-anesthesia care unit in stable condition on a non-rebreather. On the same day, the patient then switched to bilevel positive airway pressure (BiPAP) via a respiratory therapist, at which point the arterial line and the chest tube were removed. On post-op day 1, the patient stated her neck was sore by where the central line is, otherwise her pain is well controlled, no shortness of breath or other complaints. Chest X-ray was done on the second day and it was stable with no traumatic pneumothoraces and no air leak was seen. The patient was allowed to be discharged and given a follow-up appointment. Three weeks later, the patient was seen. She was stable, her incisions were well healed, and had no complaints.

Pathologic findings 

**Figure 2:**
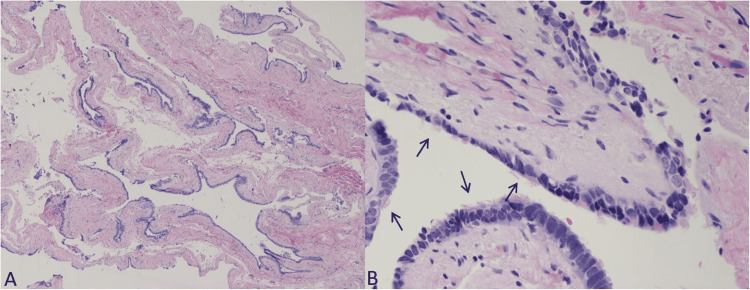
Histologic features of mediastinal Müllerian cyst. A: Cyst wall composed of fibromuscular tissue, 40x hematoxylin and eosin (H&E) stain; B: Cyst wall lined by pseudostratified columnar ciliated epithelium (arrows), 400x H&E stain

A gross pathologic examination of the specimen revealed a tan-gray disrupted saccular fragment of fibromembranous tissue, measuring 2.0 cm x 1.0 cm x 0.2 cm. Histologic sections with hematoxylin and eosin staining demonstrated a cyst wall lined with pseudostratified columnar epithelial cells resting on thin fibromuscular tissue with some of the lining cells appearing ciliated (Figure 2). 

**Figure 3:**
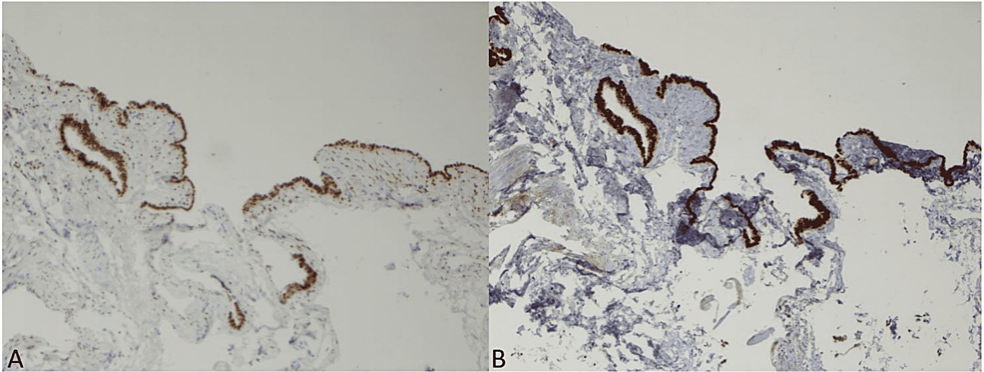
Immunohistochemistry for mediastinal Müllerian cyst lining epithelial cells. A. Immunohistochemical staining for ER, 100x; B. Immunohistochemical staining for PAX-8, 100x ER, estrogen receptor

There were no gastrointestinal features or goblet cells present. Immunohistochemical stains were performed and the cyst lining epithelial cells showed diffuse nuclear positivity for estrogen receptor (ER) and PAX-8 (Figure 3). These findings are consistent with a mediastinal Müllerian cyst.

## Discussion

Retrospective review

Materials and Methods 

After institutional review board (IRB) approval, the Geisinger Medical Center pathology database was queried for posterior mediastinal lesions for dates ranging between January 1, 1980 and June 11, 2018 (38.5-year period). H&E and immunohistochemical stains, per institution protocols, were performed as needed at the time of initial pathologic examination. All of the queried posterior mediastinal lesion reports were reviewed and select slides were reviewed for diagnostic confirmation. Immunohistochemical stains ER, progesterone receptor (PR), D2-40, WT-1, CD31, CDX-2, and TTF-1, and PAX-8 were performed and ordered to determine whether or not these cases expressed receptors consistent with Müllerian differentiation.

*Results* 

Some 135 posterior mediastinal lesions were identified within the database. Hematologic malignancies and metastatic carcinomas were excluded. Seventeen posterior mediastinal lesions were schwannoma/neurilemmomas. Seven cases, including the current case, were cystic posterior mediastinal lesions, including bronchogenic cyst, enteric cyst, lymphangioma, and non-specific benign cyst. All seven cases were re-reviewed to confirm the prior diagnoses. The demographic and diagnostic information is provided in Table 1. All seven of the cystic cases were benign. 

**Table 1:** Institutional cystic mediastinal cases.

Sex	Diagnosis
M	Bronchogenic cyst, posterior mediastinum
F	Cystic mass, posterior mediastinum
M	Benign cyst, posterior mediastinum
F	Pericardial cyst, posterior mediastinum
F	Benign cyst, posterior mediastinum
M	Cystic lymphangioma
F	Müllerian cyst, posterior mediastinum

Of the seven cases, the only one that demonstrated findings consistent with Müllerian differentiation as described within the literature was the current case. No previously diagnosed case within the last 38.5-year period that was notable for cystic components within the posterior mediastinum was found to have Müllerian differentiation. 

Discussion

In 2005, Hattori was the first to describe posterior mediastinal cysts with Müllerian differentiation [7]. A review of the existing literature began with searching for publications since January 2005 that included the terms “posterior,” “mediastinum,” “mediastinal,” “cyst,” and “Müllerian.” Boolean searches for these terms took place on databases including PubMed and Google Scholar. The results were then narrowed to include only reports translated into English. The search revealed 24 authors who reported a case of Müllerian cyst across 39 patients. This case report brings the total number of recognized patients within the literature to 40 [7-31]. All reported cases were female, with an average age of 47.2 years and a standard deviation of 8.6 years. Of the 39 individual cases in which symptoms were described prior to further diagnostic measures, 19 were termed “asymptomatic” [7-10, 14-15, 18-20, 25-30], with others describing symptoms such as “cough,” “chest pain,” “dysphagia,” and “dyspnea.” Out of the 39 reported cases of Müllerian cysts, all the cases occurred exclusively in female patients, with an average size of 30.4 mm and a standard deviation of 11.5 mm (Table 2). 

**Table 2:** Some of the reported cases in the literature. R (right-sided), L (left-sided), Th (thoracic),  "-" (data unavailable within report)

	Year	Sex	Age	Symptoms	Location		Size (mm)
Hattori	2004	F	18	Asymptomatic	R	Th5	33
	2005	F	52	Persistent cough	R	Th6	20
		F	49	Cough	L	Th4	20
Thomas-de-Montpreville	2007	F	40	Chest pain, dysphagia	L	Th4	15
		F	46	Cough	L	Th4	33
		F	47	Cough	R	Th4-5	50
		F	48	Asymptomatic	L	Th5	30
		F	50	Chest pain	R	Th3-4	32
		F	51	Asymptomatic	L	Th3-4	30
		F	56	Asymptomatic	L	Th8	13
		F	58	Cough	Prevertebral	Th5	45
		F	59	Chest pain	R	Th2-4	25
Businger	2008	F	54	Asymptomatic	L	Th4-6	45
Batt	2010	F	41	Chest pain	L	Th6	21
Kobayashi	2012	F	53	Asymptomatic	R	Th5	20
Dakak	2012	F	51	Dysphagia	"-"	Th5	27
Liao	2012	F	48	Chest pain	R	Th6?	51
Simmons	2013	F	52	Shortness of breath	R	Th6?	41
		F	47	Asymptomatic	L	"-"	50
Takahashi	2014	F	47	Asymptomatic	R	Th4-5	20
Lee	2014	F	42	Asymptomatic	R	Th6-7	26
Chon	2015	F	51	Asymptomatic	L	Th6	30
Skancke	2015	F	35	Cough	Multiple	Multiple	Multiple
Chandra	2017	F	52	Chest pain, shortness of breath, numbness of arm	L	Th3-5	48
Weedle	2017	F	37	Chest pain	L	"-"	"-"
Mowad	2017	F	49	Cough	"-"	"-"	"-"
Oshima	2017	F	48	"-"	"-"	"-"	31
	2017	F	40	"-"	"-"	"-"	"-"
Łochowski	2017	F	53	Asymptomatic	R	Th4	30
Yasukawa	2018	F	41	Asymptomatic	L	Th10	30
Idaewor	2018	F	56	Cough, shortness of breath	L	Th3-4	30
Miura	2018	F	50	Cough	L	Th6-7	19
		F	52	Asymptomatic	R	Th3-4	52
		F	46	Asymptomatic	R	Th4-5	41
		F	52	Asymptomatic	L	Th1-2	30
Sekimura	2018	F	40	Asymptomatic	L	"-"	12
Chao	2018	F	49	Asymptomatic	R	Th5	22
Lee	2018	F	22	Asymptomatic	L	Th10	24
Tsai	2018	F	44	Asymptomatic	L	"-"	18

A differential diagnosis of mediastinal cystic lesions can be generated based on a three-compartment mediastinal anatomic model [31]. The differential diagnosis of cysts that can be found in the anterior mediastinum include thymic cysts, cystic teratomas, germ cell tumors, and lymphatic malformation; the middle mediastinum includes pericardial cysts and foregut duplications; the posterior mediastinum includes lymphatic malformations, myelomeningoceles and neural derived lesions, and foregut duplication cysts [4, 32]. Due to their increased recognition within the literature, it is important to consider Müllerian cysts when encountering cystic findings of the posterior mediastinum [7, 13-14, 30].

Several theories have been proposed for the development of mediastinal Müllerian cysts. Ludwig’s theory proposes that the “thickening of the coelomic epithelium develops on the cranial end of the plica mesonephridica at the level of the third to fifth thoracic vertebral blastemal and forms the anlage of the funnel area of the fallopian tube” [33]. This would explain why most mediastinal Müllerian cysts are found between the level of T3 and T5 [5]. However, it does not account for those found outside of that range, with some being found as low as T10 [26, 29]. Hattori, who first identified this pathology suggests that the finding itself could represent misplaced mesothelium and mesenchyme with Müllerian characteristics [7-8]. Müllerian cysts located in the posterior mediastinum do not have endocervical differentiation, unlike those found in the retroperitoneum [18, 34]. The presence of cysts at distant sites outside of the pelvis has been theorized to be a result of vascular or lymphatic dissemination [26]. Lauchlan described a hypothesis that the Müllerian tissue was distributed in a centrifugal manner from the ovaries and was not limited to simply just the pelvis [35]. He showcases Müllerian tissue implantation due to metaplasia of ectopic peritoneal cells into secondary Müllerian epithelium [26]. Most of the reported mediastinal Müllerian cysts within the literature behaved in a benign fashion with no recurrence, and the effective treatment was surgical excision [1].

## Conclusions

We have reported a case of posterior mediastinal Müllerian cyst with a review of the literature and summary of the reported cases in addition to an institutional retrospective review. Any type of paravertebral findings in females, symptomatic or otherwise, should include Müllerian cysts within the differential. Furthermore, any ciliated cyst found within this region should be considered a Müllerian cyst until shown to be otherwise. This was the first case of posterior mediastinal Müllerian cyst within our health system. Unfortunately, due to the limited number of cases within the literature as well as lack of documented follow-up, there exists limited information to determine long-term clinical outcome and how these cysts might differ from bronchial or esophageal cysts. Nonetheless, it is important to consider Müllerian cysts when encountering cystic findings in the posterior mediastinum in order for patients to be counseled, managed and followed appropriately.
